# Challenges, Coping and Resources: A Thematic Analysis of Foreign Teachers’ Experience of Cultural Adaptation in China

**DOI:** 10.3389/fpsyg.2020.00168

**Published:** 2020-03-03

**Authors:** Su Yi, Ning Wu, Xiaoqin Xiang, Liang Liu

**Affiliations:** ^1^Sun Tree Counseling Center, Wenzhou-Kean University, Wenzhou, China; ^2^Shanghai Pudong New Area Mental Health Center, Tongji University School of Medicine, Shanghai, China; ^3^Department of Clinical Psychology, Shanghai East Hospital, Tongji University School of Medicine, Shanghai, China

**Keywords:** cross-culture adaptation, foreign teachers, Chinese university, coping strategies, resources

## Abstract

The aim of this study was to explore foreign teachers’ experience of cultural and life adaptation in China, including the difficulties, emotional stress, and challenges they confronted, as well as the useful coping strategies and resources that facilitated their cultural adaptation. Ten foreign professors who taught in China were recruited from three Chinese universities. Semi-structured interviews on the participants’ experience of cross-cultural adaptation in China were conducted. Thematic analysis of the transcribed dialogs was performed, and five core themes emerged: *multiple challenges*, *mixed negative emotions*, *active coping and insulation*, *rich supportive resources*, and *personal traits*. In the cross-cultural adaptation process in China, the main challenges for foreign teachers were language barriers; a lack of supportive relationships; and conflicts in perceptions related to teaching, privacy, and boundaries. Useful coping strategies included seeking interpersonal interactions that provided emotional and instrumental support, developing localized teaching models, and maintaining connections with the culture of origin. Under circumstances with convenient living facilities and supportive interpersonal relationships, these strategies promoted the adaptation of foreign teachers with functional personality traits. The potential correlations among these different themes and the influence of their interactions on foreign teachers’ adaptation experience were also discussed. The themes that emerged in our study can help clinicians and university administrators develop therapy methods and support systems for foreign teachers who work in Chinese universities.

## Introduction

With the constant development of the Chinese economy and against the background of globalization, an increasing number of foreign teachers are coming to China. The number of foreign teachers in China reportedly reached 157,076 in 2015 (with most of them coming from America and Europe) and has been increasing over the past few years, especially in Chinese universities (Cui et al., 2016; Meng, 2017). The importation of foreign teachers has improved the diversity and internationalization of teaching methods and content in Chinese schools and has promoted the exchange of Chinese and foreign cultures and academics.

However, the recruitment of more foreign teachers in eastern Asia has also been accompanied by challenges for these foreign educators related to their daily lives and cultural adaptation. For example, some prior cross-sectional studies used self-designed quantitative inventories to assess the daily challenges faced by foreign teachers in China. They found that the challenges foreign teachers regularly faced were complicated and diverse and included language barriers; differences in diet, living habits, and weather; conflicts in cultural perspectives of interpersonal boundaries; conflicts regarding teaching methods and models; problems with the administration patterns of Chinese schools; and feelings of loneliness and isolation, homesickness, and culture shock (Ma, 2007; Wang, 2010; Duan, 2012; Ilham, 2015; Cao, 2017). Similarly, Melby et al. (2008) adopted a phenomenological approach to explore the lived experience of Western nurse educators teaching at an eastern Asian university. They found that language difficulty, cross-cultural differences in pedagogies, and conflicts between collectivism and individualism were the main stressors for the subjects (Melby et al., 2008).

Meanwhile, previous literature has also indicated diverse emotional and cognitive responses of foreign teachers who confront such challenges. For instance, McKinnon et al. (2014) quantitatively investigated the self-efficacy of foreign teachers who taught science in Abu Dhabi’s primary, private, and public schools. They found that both beginning and experienced teachers showed a low sense of self-efficacy and a lack of a sense of strength (McKinnon et al., 2014). Moreover, the study by Melby et al. (2008) also implied that, for English-speaking nurse educators who taught in eastern Asia, emotional reactions such as exhaustion and confusion were effects that could accompany their cultural adaptation processes.

The results of the above studies, although mostly based on quantitative surveys lacking exploration of the vivid and subjective experiences of participants, provided a general framework for understanding the daily problems of foreign teachers who teach in a culture that is not their own. Meanwhile, despite very limited information, these previous studies also provided preliminary insight into foreign educators’ corresponding emotional reactions and perspectives. However, this information is still not sufficient for the construction of a complete, systemic understanding of the mechanism of foreign teachers’ cultural adaptation. According to previous research on cultural adaptation among other populations, other potential factors affecting foreign educators’ cross-cultural adaptation may include the effective or dysfunctional coping strategies they adopt, variables related to their personal traits and flexibility, and variables related to their social and family environments (An and Chiang, 2015; Saffari et al., 2015, 2017).

The influence of such factors on foreign teachers’ adaptation to and assimilation into a different society remains underexplored. Although some scholars have tried to examine the strategies subjects adopt to cope with stress due to cross-cultural adaptation, their results have been based more on individual case reporting rather than on an empirical analysis of the subjects’ experience. For example, Meng (2017) shared the stories of three foreign teachers at a university in China to explore their emotional self-regulation mechanisms during cultural adaptation. Their results implied that active coping behaviors, with respect to Chinese local culture, timely observation, and necessary emotional isolation could promote better adaptation to the Chinese environment. However, their results were not derived from empirical data, which limited the generalization of their conclusions to a larger group.

With respect to the potential personal and environmental factors affecting foreign teachers’ cultural adaptation, some researchers have shared subjective perspectives based on their experience communicating with foreign teachers. The ideas they have proposed are diverse and complicated. For instance, based on the experience of daily interactions with foreign teachers who worked in a Chinese university, Li (2012) proposed that teachers’ previous cross-cultural experience and positive information from the media have positive effects on individual cross-cultural adaptation. Li also emphasized that foreign teachers’ optimistic attitude toward stress and their flexibility in terms of their cultural identity play important roles in their cultural adaptation strategies. Similarly, Meng (2017) proposed that spiritual support from intimate others and a flexible, outgoing personality might facilitate foreign teachers’ adaptation. Moreover, a quantitative investigation of foreign teachers from different levels of schools in Tianjing suggested that being married, being accompanied by one’s family, living in China for more than 1 year, not being in China for the first time, and having good social support could improve foreigners’ adaptation processes (Cui et al., 2016). Nevertheless, these studies were limited to the researchers’ subjective experiences or quantitative assessments and lacked a detailed exploration of participants’ experience. Hence, the usefulness and implications of their findings are very limited, and caution should be exercised when extending these ideas to apply them to other groups of people.

A review of the limited literature on foreign teachers’ adaptation to a new culture indicates several gaps. Firstly, most previous studies provided only simple phenomenological descriptions of the cross-cultural difficulties that foreign teachers confront and their emotional responses and were based on quantitative surveys without an in-depth examination of foreign teachers’ subjective experiences. Secondly, with respect to the potential factors affecting foreign teachers’ cultural adaptation and the coping strategies that they adopt to deal with daily challenges, prior literature has been limited to the subjective experiences of the researchers, case reports, or quantitative surveys and lacked an in-depth exploration of the participants’ own experience using an empirical approach. Thirdly, most prior studies focused only on a single area of foreign teachers’ cultural adaptation without threading different aspects, such as the challenges they experience, their emotional responses, their methods of coping and the factors that facilitate or hinder their adaptation, and their potential interactions, into a complete story. Fourthly, the only phenomenological analysis of foreign educators’ experience of adaptation in Asian countries was restricted to the discipline of nursing. The subjective experiences of foreign teachers who work in other specialties remains unexplored. Lastly, most previous research has explored the adaptation process without distinguishing foreign teachers teaching at universities from those working in primary and middle schools. This oversight affects the targeted application of the results to the corresponding population.

Hence, to address the research gaps mentioned above, the current qualitative study explored the subjective experiences of foreign teachers working at Chinese universities in terms of the difficulties, emotional stress, and challenges they faced during the process of cultural adaptation in China. Special attention was also paid to the useful strategies that subjects used to cope with these challenges and the potential factors, such as their personal traits, environmental variables, and possible resources, affecting their adaptation processes. In other words, based on an empirical and qualitative approach, we aimed to create a more comprehensive picture of foreign teachers’ cultural adaptation experience by incorporating the diverse dimensions mentioned above.

## Materials and Methods

### Sample

From March 2019 to May 2019, 10 foreign professors teaching in China were recruited from three universities in Zhejiang Province and Shanghai. The sample inclusion criteria were as follows: (1) holds a formal teaching position at a university; (2) has no diagnosed medical or surgical condition or mental disorder; (3) is not a native speaker of Chinese; and (4) has not lived in China for an extended period. All the subjects participated in this study voluntarily. See Table 1 for the demographic information of the participants. To protect the privacy of the subjects, we use “P1, P2… P10” to represent the different subjects when quoting their conversations.

**TABLE 1:** Demographic information of the subjects.

No.	Gender	Age range	Country of	Years working	Intimate relationship/	Number of	Family member or	Position	Disciplinary	Past experience
		(years)	origin	in China	marital status	children	relationship in China		areas	living abroad
P1	Male	75	United States	1	Yes	2	No	Dean	Science and Technology	Asia
P2	Male	52	United States	4	No	0	No	Lecturer	Mathematic	None
P3	Male	66	United States	3	Yes	5	Yes	Assistant Professor	Visual design	Asia and Europe
P4	Male	43	United Kingdom	5	No	0	No	Lecturer	English teaching	None
P5	Male	58	United States	4	No	2	No	Assistant Professor	Biology	None
P6	Male	57	Canada	1	Yes	0	Yes	Professor	Psychology	Asia and the USA
P7	Female	49	United States	1	Yes	0	No	Lecturer	General education	None
P8	Female	29	Russia	4.5	Yes	0	Yes	Lecturer	English	None
P9	Male	54	United States	5	No	2	No	Professor	Psychotherapy	Asia
P10	Female	37	United Kingdom	3	Yes	3	Yes	Assistant Professor	Medicine	Asia

### Procedure

We released the subject recruitment advertisements through the university Bulletin Board System (BBS) and administrative offices of diverse colleges. Foreign teachers who were willing to participate in the study contacted the researchers by e-mail or phone. Then, the purpose, content, process, and principle of confidentiality of the study were introduced to the potential participants in accordance with standardized instructions. For foreign professors who decided to participate and met the inclusion criteria, the researchers first invited them to the research office. Their future questions were answered, and their willingness to participate was confirmed again. Then, the participants signed the formal informed consent form, and the researchers then made an appointment for a formal interview. The research proposal received ethical approval from the ethics committee of the universities that were responsible for the management of this study.

The interview was a semi-structured one in which the interviewers asked the subjects questions according to a predesigned outline. The interview outline was designed according to the topic of this research, i.e., foreign professors’ adaptation and coping in China. The interviewers tried their best to ask open questions during the interview and made necessary inquiries to clarify any vague answers, such as “Could you tell me more?” or “What exactly does that mean?” In addition, few comments or guiding statements were made to ensure the continuity and spontaneity of the interview process. The interview outline was as follows: (1) Please describe the challenges you have experienced since you began teaching in China. If possible, please provide some details. (2) How did those challenges affect you at that time? If possible, please provide some details. (3) How did you handle that situation? Please detail the issue and evaluate your solutions from your current perspective. (4) Were there any people or resources that supported you in that situation? (5) If you encounter similar challenges in the future, what could be some potential resources for you?

The formal interview time for each subject varied from 1 to 1.5 h, and the interview was conducted in a separate, quiet, and confidential room. The entire interview process was recorded with a digital recording pen. The interviews were completed by the second and third authors of this paper, who both have received at least 3 years of formal social psychology research training and have 2–4 years of psychological counseling experience. Both are fluent English speakers; hence, there were no language barriers during the interview.

### Data Analysis

Thematic analysis was used to analyze the transcribed data following the guidance provided by Braun and Clarke (2006). This approach allowed us to interpret the meaning of the data and process complicated data to discover patterns. The data analysts were the four authors (one male and three females) of this paper, all of whom have a doctorate or a master’s degree in the field of psychiatry or social psychology. All of the analysts are English speakers who do not have difficulty in English communication and understanding. Prior to the initiation of this study, all four investigators had 2–10 years of clinical training in counseling and at least 1 year of qualitative research experience, with a maximum of 10 years of experience. Meanwhile, 4 months of training was also conducted on the research topic, analytical methods, and guiding ideology before the study began. The analytical procedure consisted of the following phases.

#### First Phase: Familiarization With the Data

Verbatim transcriptions of the digitally recorded interviews were conducted by the researchers. Then, the researchers read through the transcribed materials repeatedly. The accuracy of the transcriptions was checked. Meanwhile, 4 months of training was conducted on the research topic, analytical methods, and guiding ideology. This process also encouraged the researchers to immerse themselves in and become familiar with the data.

#### Second Phase: Initial Code Generation

During the second stage, each interview transcript was reread. Then, initial codes identifying a feature of the data (semantic or latent content) that appeared relevant to the research topic, i.e., foreign professors’ adaptation and coping in China, were developed and assigned. These codes referred to the most basic segment or element of the data that could be assessed in a meaningful way regarding the research topic. The initial codes were added to any word, sentence, or paragraph that the researcher considered noteworthy. The initial codes are shown in Table 2.

**TABLE 2:** Core themes, subthemes, and initial codes.

Core themes	Subthemes	Codes
*Multiple challenges*	*Language and daily life barriers*	Language, daily life, life difference, different etiquette or custom, cutting into the line or spitting, foreigner role, cumbersome banking process
	*Differences in teaching concepts and methods*	Different teaching or thinking method, different teaching concepts
	*Closed-loop social interaction patterns and lack of connection*	Different ways of building interpersonal relationships, the emphases of family relationship, lack of interpersonal connection, lack of proper counseling
	*Different understanding of privacy and boundaries*	Freedom of speech, privacy, got extra attention or charged more as a foreigner, different administrative ways, leader would limit his subordinates to talk
*Mixed negative emotions*	*Frustration*	Frustrated, sad, depressed, stressful, guilty
	*Embarrassment*	Embarrassed, shamed
	*Anger*	Angry, irritated
*Active coping and insulation*	*Functional interaction*	Ask for help from Chinese people (Chinese colleagues colleges/friends/students), pay attention to possible resources when interacting with students, actively interact with people in order to cope with loneliness, keep clear boundaries between work and life to protect privacy, seek counseling, change the system through own actions
	*Localization*	Use popular apps, use integrated teaching method, maintain a fixed lifestyle, observe things in detail, develop new skills
	*Emotional insulation*	Insulate emotions, insulate needs, inefficient coping way, not using smartphone
	*Keeping in touch with the culture of origin*	Maintain familiar habits, keep in touch with compatriots, speaking in mother tongue
*Rich supportive resources*	*Convenient app and facilities*	Convenient mobile phone software, Western restaurants, imported supermarkets
	*Help from local agencies and people*	Life support from workplace, help from people who know Chinese (friends, colleagues, graduate students), foreigner status
	*Multicultural competency and life experience*	Multicultural life experience, cross-cultural teaching experience, rich life experience
	*Stable intimate relationships*	Stable intimate relationships
*Personal traits*	*The trait of positive thinking*	View problems as challenges and opportunities, expectations for intimacy, love for China, love of living in the moment
	*Avoidance coping*	Suppress emotions, avoid relationships, deny needs
	*Low flexibility*	Easy to produce a sense of shame, refuse to use smartphones, high sensitivity of food quality, physiological characteristics, cleanliness

#### Third Phase: Search for Themes

After obtaining the initial codes, the researchers repeatedly compared the primary codes with the text content and with each other. Conceptually related and similar codes were sorted into higher-level themes, and all the relevant coded data extracts within the identified themes were collated. As suggested by Braun and Clarke (2006), a thematic map was developed to aid the identification and formation of the overall themes by the grouping initial codes.

#### Fourth Phase: Review of the Themes

This phase involved checking and refining the candidate themes against the coded data extracts and the entire data set. The researchers made comparisons between the candidate themes in accordance with the dual criteria for judging categories: *internal homogeneity* and *external heterogeneity*. Themes without sufficient data were abandoned. Overly complicated themes were divided into multiple themes, and similar themes were merged into each other. When the candidate themes adequately captured the contours of the coded data, the validity of the individual themes was considered in relation to the entire data set to determine whether the themes reflected the meanings evident in the data set as a whole and told a convincing story of the data. At the end of this phase, saturation of the themes was also reached.

#### Fifth Phase: Definition and Naming of the Themes

During this stage, the researchers defined and refined the themes by returning to the collated data extracts for each theme and organizing them into a coherent and internally consistent account with an accompanying narrative. A detailed analysis of each theme was conducted, and the scope, definition, and story of each individual theme were determined. Any potential subthemes were also identified. The title of each theme was designed to be concise and effective to immediately convey the meanings of the themes. This phase of analysis was recursive until the final theme map could build a complete story that was consistent with the research objectives.

#### Sixth Phase: Report Production

This final phase involved organizing the fully developed themes and corresponding data extracts and writing up the reports. The themes were woven together with the analytic narratives and most representative quotes that could capture the essence of the themes. Finally, a report that could illustrate the whole story of the data was completed.

### Credibility and Dependability of Data Analysis

For qualitative studies, analysts themselves are tools of the research. The impact of their biases on the research process and results should not be ignored. To bolster the study credibility and dependability, research meetings were held for at least 2 h every week during the entire research process. Any questions, confusion, and ideas about the study were discussed and resolved in a timely manner. Especially during the data analysis stages, the codes and themes that any one analyst produced were regularly reviewed by the other three researchers. Meanwhile, the emerging themes were reviewed by the four analysts together during every research meeting. Any divergence of opinions relevant to the coding, searching, definition, and naming of themes were fully discussed. Finally, consensus on the results, including the final thematic map presented here, was achieved.

## Results

**FIGURE 1:**
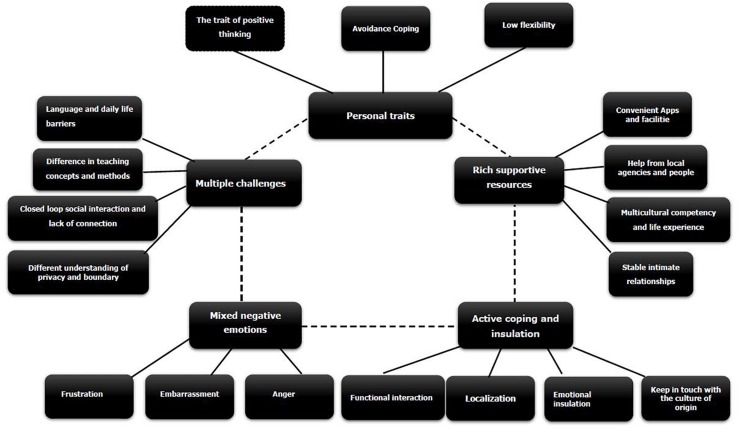
Map of themes derived from initial codes.

Five core themes emerged from our analysis—namely, *multiple challenges*, *mixed negative emotions*, *active coping and insulation*, *rich supportive resources*, and *personal traits*—among which, there were 19 subthemes (see Figure 1). The specific analysis results were as follows.

### Theme 1: Multiple Challenges

Participants mentioned that they experienced language barriers while living in China, as well as conflicts between their native culture and Chinese living habits, cultural concepts, teaching work, interpersonal communication customs, and ways of thinking, which introduced challenges to their adaptation processes in China. Specifically, these challenges included language and daily life barriers, conflicts about teaching philosophies and methods, closed interpersonal communication modes and loneliness, and different understandings of privacy and boundaries.

#### Language and Daily Life Barriers

All 10 participants reported that the most common challenge they encountered while living in China was the language problem. They encountered difficulties in adapting to the language problem in their daily work, teaching, and life, as the following examples demonstrate:

“*We are almost all of us illiterate. So, we cannot hear, we cannot speak, we cannot read. So, I have a cognitive shutdown visually because of signs that don’t have Arabic characters*.” (P7)

“*(A cashier) just asked me, you know, do I need a bag? But of course, it was in Chinese, so I didn’t know what she was asking me, and I kept looking for the bags at the end of the counter*.” (P2)

There were also differences in daily life, such as the cumbersome procedures of banks or even the toilet facilities, which made them feel uncomfortable, as the following quotations show:

“*This should be transparent to the bank on this, because a Chinese group can transfer money just by going online; a foreigner, you have to go, and you have to prove your final balance, and they will not say that sometimes you can, you do not need to do that, and they will force you, or they will recommend you to do all the same things every time when you are trying to change some things that are not the same thing*.” (P4)

“*The toilets, I mean, the holes in the floor, I can’t do; that is a cultural thing, I understand, so you know, whenever I go places, I will ask, “Is there a sitting toilet?*” (P3)

#### Differences in Teaching Concepts and Methods

The conflicts about teaching concepts and methods mainly focused on the following aspects. In terms of teaching methods, both P4 and P1 thought that China attached more importance to English reading and writing while ignoring listening and speaking.

“*I think that’s also challenging because the level of (English) listening is really one of the problems in China. People don’t really teach listening to the same degree because there’s a focus on reading and writing. When you stand in a class as a native speaker trying to communicate to students in English, you realize that they don’t really get 80% that is beyond their understanding*.” (P4)

In addition, P3, P1, and P2 all mentioned that Chinese students were accustomed to silence in class, which was quite different from the teachers’ original style of frequent teacher–student interaction and inspiring students to think through discussions.

“*One of the things that I was not prepared for was how quiet the students are, and so when you ask a question in the kind of traditional education fashion, we wait for an answer, but the answer does not come*.” (P3)

There were also some differences in the understanding of teaching concepts. Traditional Chinese teaching emphasizes that teachers should follow the whole teaching program designed by the teaching committee and management department of the university. This teaching style conflicted with the teaching concepts of some interviewees, who thought that teaching plans should respect teachers’ own wishes.

“*Let me think about it in the concept of teaching. When I first came to China, I only had a small introduction to the idea of teaching. I first thought that their wishes (those of the management department of the university) were secondary to the requirements of the course. But now, it’s different. You should follow their instructions and plan. You are just an addition to the university*.” (P4)

#### Closed-Loop Social Interaction Patterns and a Lack of Connection

The interviewees experienced certain cultural differences in the way interpersonal communication occurred. Most Chinese people like to communicate with people they are familiar with. P4, P8, and P9 all mentioned this relatively closed communication mode, which was difficult to adjust to when they first came to China.

“*I knew I needed to create a social network that wasn’t closed, because often that was what was happening here. And when I first came, I had a friend, and groups were brought into a friend group, and always, maybe I could call it the KTV phenomenon, your friends and you go to KTV, and nobody else comes into that group. So, unless someone is invited in from some other thing, then that group is quite closed. And I felt that there was this very closed culture within ordinary Chinese socializing*.” (P4)

The ways of building relationships mentioned above also made new teachers in China feel disconnected, lonely, and frustrated. Some subjects even felt a lack of control over their interpersonal relationships and a low sense of self-worth, as P8 mentioned.

“*At the beginning, I didn’t really feel connected with my colleagues. I really had this huge gap in communication between me and my colleagues. I just felt lonely and, hum, frustrated. I even sometimes wondered if I was worthy of being recognized and associated with others. I felt like I was losing the internal and external locus of control*.”

Some participants described the way that Chinese people, including their colleagues, attach great importance to parent–child and family relations. The way that Chinese people socialize with family and friends also made them feel different and awkward.

“*(I think) friends are more important than family when you become an adult, but in China, it has been the complete opposite. People obviously take families to be closer and, hum, much more important. You know, it looks like a closed loop. No matter what they do, the first groups to consider and trust are families and acquaintances, even if sometimes a friend or colleague may be the better choice. But most of the time, people would take the places (of their friends), but it was always their kids or relatives*.” (P9)

#### Different Understandings of Privacy and Boundaries

Some participants felt that boundaries regarding the respect and control of privacy in China were different from those they were used to in their native cultures and mentioned behavior such as taking and sending photos of others without the permission of the party concerned, being followed and lectured by others on the street, or being asked personal questions during a job interview.

“*I don’t like using WeChat (a very popular social app in China), as some of these WeChat groups are too open; it’s pretty large. There’s my picture with some other people already, and that gets sent to another group in another group*.” (P1)

“*Like interviewing candidates for jobs as new faculty members, ‘Are you married?’ You cannot ask that question. This is my personal thing, right? Personal information. ‘What race are you?’*” (P10)

In addition, in daily life, some people felt uncomfortable when their identity as foreigners was given too much attention or taken advantage of.

“*I will always be considered when I am in China as a foreigner; they will go and say “foreigner!” In the States, I would never dream of calling, if I see an Asian person, saying that they are foreigners*.” (P9)

“*I think they (the Chinese people who tried to contact the subject) are more interested in practicing English with me, yeah, just talking, but not making friends with me. Yeah, because lots of people in China are just interested in English. That’s the reason for communicating with you. Sometimes I feel like I’m being used*.” (P4)

Additionally, some subjects mentioned that, in the Chinese university administrative system, people tended to interrupt each other when communicating, and the boundaries of individual utterances were easily confused, which made them feel uncomfortable. For example, P1 mentioned,

“*You know, a high-level meeting. This person will try to dominate the conversation. We’ll try to just talk over the person who’s already speaking. And that’s where I’ll just step forward and say, “Would you please? He’s speaking, give him a chance to speak.” I’m just telling them essentially to shut up. I don’t say that; I guess I need to learn to tolerate those people better*.”

### Theme 2: Mixed Negative Emotions

In the process of adaptation in China, the participants had different levels of emotional arousal when encountering different challenges. Frustration, embarrassment, and anger were common emotions. However, it was difficult for these emotions to be expressed, understood, and responded to.

#### Frustration

Frustration was the most common emotion of the participants when they met challenges, including language and life barriers, conflicts in teaching, and interpersonal insulation. For example, P1, P2, P3, and P4 all indicated that they felt frustrated when their Chinese students did not like to speak to answer questions in class.”

“*I was frustrated for the first week, two weeks; I was trying to find ideas to get them to open up*.” (P1)

“*We wait for an answer, and if an answer does not come, then we wait some more; eventually somebody will finally answer the question, at least in country Y (P3’s motherland), but here in China, it doesn’t get answered. That’s been a little frustrating*.” (P3)

#### Embarrassment

The participants mentioned that they felt embarrassed when their status as foreigners was given undue attention or when they experienced difficulties brought about by language and life barriers. For example, P2 mentioned the following:

“*I just know that I’ll be looked at, and I just know that four hundred people are saying, ‘Foreigner,’ you know, ‘the foreigner’s here,’ and I get my picture taken sometimes*.” (P2)

#### Anger

When diverse problems and challenges could not be solved effectively, some participants occasionally felt anger. For example, P2 indicated that, when he was in class, if the students repeatedly ignored his instructions and if he tried several ways to deal with the situation but not succeed, he felt offended.

“*I find it offensive, almost personally offensive, that you are coming to class to ignore me*.” (P2)

“*That’s how I know it’s anger because it’s VPN technology, on my computer; I try it once, twice, three times, another way. It’s not because I didn’t get supported—the IT helped a lot, but it’s the fact that I have to use another Internet, virtual private network, to reach some webpages which I can easily open in other countries*.” (P7)

### Theme 3: Active Coping and Insulation

In the process of adaptation in China, finding a method of coping with challenges was common, whether it was a positive response or isolation, and helped participants to further adapt to the situation in China to different degrees. These coping strategies were functional interaction, localization, emotional insulation, and keeping in touch with the culture of origin.

#### Functional Interaction

Functional interaction refers to some participants’ efforts to have as many interactions with Chinese people as possible to seek assistance in teaching and daily life when encountering challenges in China. Some of the subjects benefited from communication with Chinese friends or students who could speak English. They helped foreign teachers become familiar with the Chinese culture and manage other Chinese students.”

“*So, I make Chinese friends who speak English, and they interpret a lot of cultural things for me; it’s through their English-speaking ability, so they help me know lots of Chinese culture*.” (P7)

In addition, P3 also mentioned that, even though some Chinese people could not speak English, they were very enthusiastic and sought to help through gestures or other means. For example, P3 related the following experience when he was in the hospital:

“*One Chinese gentleman of course talked to me in Chinese, so I gave him my business card, and he read it; when I tried to get up, he jumped up to help me to stand, because my leg hurt. Chinese people, they are very kind, very gracious. They look out for each other. They look out for foreigners*.” (P3)

In addition to improving their interpersonal relationships, some participants also made adjustments by actively accessing the interpersonal resources around them. For example, P7 mentioned that, when she felt lonely in China, she joined an online cooking group.

“*I can trust their food, because that chicken is more trustworthy; it can help me to deal with my frozen chicken and shrimp. The online group supported me a lot, not only in the cooking part but also, more importantly, the connection with other people*.”

Some participants mentioned that, when they felt depressed or heartbroken, they would seek help from local psychological counselors.

“*So, all, everything that I was feeling, like all this negativity, all this, I just talked it out; you find a psychologist, and you talk with that person*.” (P8)

### Localization

Localization refers to participants’ gradual alignment of themselves more with China’s national conditions and their adoption of local methods to improve a situation or resolve problems associated with teaching or life challenges they faced in China. For example, in dealing with the conflicts regarding teaching concepts and methods, P3 and P4 made necessary adjustments to the interactive mode of their classes, the grade evaluation system, teaching multimedia, and other aspects to bring them more in line with the habits of Chinese students, such as by taking the initiative to call roll, linking grades to classroom speech acts, and using video in their teaching. As mentioned by P3,

“*Any students will not respond to a stranger; they will not invest in you unless you invest in them, so I get their names, I tell a lot of jokes, I explain it to them, and I get a couple of chuckles*.”

“*Television covers interesting topics, so it will already contain complex topics. So, they’re also getting introduced to new language environments*.” (P4)

In addition, P2, P3, and P6 all mentioned that they handled language problems by maintaining certain fixed lifestyles, such as by taking the same bus at the same time and going to the same restaurant.

“*I get on bus X (pseudonym for the bus number) and go to market X (pseudonym for the market). See? No problem. Two and a half yuan from, um, I always have the card where I want to go so I won’t get lost or confused*.” (P3)

Some participants (P1–P3) solved difficulties by downloading software and apps that made life in China easier.

“*How you, you know, when you go to make, say you need to like, buy something, but they (the products) are all labeled in Chinese; I’ve got another app. Maybe it’s our no, no. Um, um, it’s that one also where there is a camera, ah, so you can scan, and I can take a picture of it or scan it, and it comes up in English*.” (P3)

Several foreign teachers solved their language problems by looking more carefully at the appearance of products in supermarkets and being sensitive to new information in their surroundings.

“*Even this is a challenge, just, I have to look really closely and realize, oh, that is what I’m looking for. I just didn’t know. And I mean of course, I have to rely on what I’m seeing because I can’t read the description. They are all in Chinese*.” (P2)

### Emotional Insulation

In addition to the positive responses, some participants, such as P5, P1, and P6, said that, when problems could not be reconciled, the appropriate suppression of their own needs to accept their reality was an important way to adjust their physical and mental states.

“*I see maybe some foreign teachers, and they’re acting, they’re degrading the people there, complaining about some things being dirty or you know, just shut up, don’t say anything. This is culture; this is the way it is, except if you don’t like it, don’t come here. Go home and enjoy your little apartment to live in, but don’t say anything*.” (P1)

However, for some participants, it seemed that excessive suppression of their emotions or needs for interpersonal connections stood in the way of their adaptation.

“*When I feel lonely, I usually just have these internal conversations with myself. So yeah, I usually think I just internalize a lot of a lot of things without thinking too much*.” (P2)

“*People speeding or you know, really, I cannot do anything, I just close my eyes because it makes me vomit*.” (P6)

#### Keeping in Touch With the Culture of Origin

The participants mentioned that staying connected to their native cultures and customary living habits helped them better adapt to living in China. For example, they tended to retain their customary patterns of making friends, cooking at home, and communicating with people from the same country. These connections helped them build a relative sense of continuity and promoted their adaptation to their new lives in China.

“*I realized that in community groups, I created an English corner. And because I had an English corner, British people then who all spoke English, I created this social network. I knew I needed to create a social network that wasn’t closed, because often that is what was happening*.” (P4)

“*I know the store, and I also learned how to bake my own bread. Whenever I have time, I bake my own bread at home. I am a picky person, not the kind of person who would eat everything*.” (P6)

### Theme 4: Rich Supportive Resources

During the process of adapting to life in China, six of the participants (P1, P3, P4, P6, P9, and P10) actively or passively solved problems themselves using software and the surrounding facilities and local or cross-cultural resources. There were four subthemes: convenient apps and facilities, help from local agencies and local people in China, multicultural competency and life experience, and stable intimate relationships.

#### Convenient Apps and Facilities

P4 and P6 mentioned that there were many Western-style supermarkets and restaurants in China, such as KFC and McDonald’s, where participants could buy food and daily necessities that suited their own tastes and habits.

“*I go to supermarket O (pseudonym for an import supermarket) because that chicken is more trustworthy. I can trust their food. Their whole wheat, those dark breads. So, I’ll buy those because that’s all I eat*.” (P4)

Several participants mentioned that with smartphone apps, it was very convenient to access local resources.

“*I do have this app, the kind where I can talk in English, and it translates to Chinese. So that’s helped a lot, so I’ve learned that works as long as I have a signal to do it*.” (P1)

“*They had this app called Didi (a popular transportation app in China). I didn’t know why I’d never heard of it, that they put in something, and the car rolls up there*.” (P10)

#### Help From Local Agencies and People

P3, P6, P7, P9, and P10 mentioned that obtaining local resources, such as the service support provided by their work unit and help from native people who could speak English, helped them adapt more quickly. P3 mentioned that his school had a service and consultation office set up specifically for foreign professors.

“*A lot of people, when you meet challenges, and the Chinese staff in the training building, they’re the best I have ever seen. So efficient, and things get done like that. I’m just amazed. We don’t have that at university X (the university where P3 worked). So, it’s been wonderful*.” (P3)

In other cases, when participants had difficulties in daily life, the related agencies would provide active assistance quickly.

“*The landlords wouldn’t even care to fix things in the apartment, so it’s our university that sends the repair person to the apartment to fix things*.” (P6)

In addition, it was very helpful to know some local Chinese people who could speak English. As P7 mentioned,

“*They spoke English quite well; they help us with technology, they help us with food advice, they help us shop*.”

#### Multicultural Competency and Life Experience

Some of the participants had many successful experiences in life and teaching. They thought that these rich life experiences had promoted their cultural adaptation process in China. For example,

“*I am the faculty with the most teaching expertise. My experience tells me (when coming into a different culture and having contact with new people) the first thing you need to do is smile. The second thing you do need to do is bring enthusiasm, have passion, and never get angry*.” (P3)

P1, P6, and P10 also mentioned that their cross-cultural experience increased their resilience as they adapted in China.

“*I am an experienced traveler, so I have been in many places in the world, so I am a little bit more adaptable and resilient to all these sort of things*.” (P6)

P1, P3, and P9 had lived or worked in other cultures before coming to China to teach. They reported that these cross-cultural experiences in Asia, Europe, and other places made their adaptation process in China smoother.

“*I was in Vietnam for over a year, and I’ve been to, I’ve been to other Asian countries. I’m not saying, um, I’m used to everything, but um, I’m accepting, I accept other cultures at equal value*.” (P1)

“*We’ve been to China before, so we knew what we were getting into. Um, and again, this is the fourth country I’ve lived in. So, we have the same thing. When I lived in Japan, we had the same thing when I lived in Europe*.” (P3)

#### Stable Intimate Relationships

Having a stable intimate relationship helped participants to better face challenges. P3, P6, and P8 were all in China with their partners. P3 mentioned that it eased his frustration to just go home and rest and have his home life.

“*When I go home, I’ve had a long day, I’m tired. Um, I either watch a little television. Usually, what happens is I could have a bite, and I just go to sleep*.” (P3)

“*My life without my boyfriend was only work, so he helped a lot. He has a different style. He is not a workaholic like me; he is more of a laid-back person and kind of drives me crazy. He does help in terms of relaxing; we are going to do something different, not just your work beloved work, but movies, hiking something else*.” (P8)

### Theme 5: Personal Traits

Different personality traits also helped or influenced the participants’ adaptation in China to different degrees. This core theme mainly consisted of three parts, namely, the trait of positive thinking, avoidance coping, and low flexibility.

#### The Trait of Positive Thinking

Some participants were full of yearning and expectations for a new life and environment. Even when they encountered different cultures and conflicts, they tended to treat such problems as challenges or differences, to actively face them and to live in the present. This coping style facilitated their adaptation in China.

“*There are no problems. We have challenges, yes. Um, and I try to tell that to students; all the time, students say, ‘We have a problem.’ I say, ‘No, you don’t have a problem; you have a challenge. We will take care of it.’ And that’s the way I see myself also*.” (P1)

“*It is more that I appreciate, began to appreciate the challenge of it; it’s an opportunity for me to practice my competence. I actually feel empowered about this*.” (P8)

Some participants loved the Chinese culture. This positive impression of China improved the participants’ experience in the country. As P5 mentioned,

“*The culture, my gosh, the culture is simply incredible; no wonder Chinese are so proud. Probably the most beautiful culture in the world*.”

#### Avoidance Coping

However, some participants, before coming to China, had been accustomed to dealing with problems by isolating themselves, which helped them avoid some conflicts, but also led to some emotional difficulties. For example, P2 and P6 mentioned the following:

“*I would not say my adaptability is great, because a lot of it is just avoidance. That is, what I worry about is I adapt by avoiding many situations*.” (P2)

“*Although I have a lot of experience traveling and living in other countries, I have to say, hum, in fact, I’m still quite used to avoiding problems. Sometimes I feel upset, but I’m not used to expressing it. Although sometimes it helps me avoid trouble, it also brings some problems, which makes me feel bad*.” (P6)

#### Low Flexibility

Maintaining one’s customary way of life may help an individual stay in touch with his or her culture of origin and promote stability, but if an individual constantly seeks stability, he or she can easily become inflexible. For example, P2 refused to use a mobile phone, even though he knew that using a mobile phone would decrease the difficulty of adapting to life in China.

“*The problem is, I don’t have a phone; I mean, I don’t have a smartphone. I am aware of some of these apps; for me, it would probably be bad because I would be even more isolated*.” (P2)

P6 was overly concerned about the safety and hygiene of food, insisting on cooking or obtaining food in his own way.

“*I don’t trust the food, like that it’s healthy, unless I know that it is, like salmon, for instance, I know salmon you have to go to the ocean to catch salmon rather than in the river*.” (P6)

## Discussion

To the best of our knowledge, this is the first study exploring foreign teachers’ cross-cultural adaptation processes in China based on a qualitative analysis of the participants’ subjective experiences. The five themes that emerged provided an initial and comprehensive theoretical framework for understanding the challenges, emotional responses, coping strategies, resources, and personal traits influencing foreign teachers’ adaptation to Chinese culture.

The first theme that emerged from the data was multiple challenges. Among the multiple challenges that teachers faced, the most common was the language barrier, which affected the daily communication and lives of foreign teachers. However, most of the participants did not learn Chinese systematically. This result is consistent with data reported in previous studies (Cao, 2017; Ma, 2007), suggesting that reasonable language training programs or realistic Chinese learning environments may need to be supplemented. Similar to the results of a previous research (Cao, 2017), our results showed that the participants in our study emphasized the difficulties created by differences in daily living habits. These conflicts caused problems when the participants first arrived in China.

Comparatively, the conflicts between Chinese closed-loop interpersonal interactions and Western individual-to-individual communication patterns seemed more troublesome for foreign teachers (Melby et al., 2008). The cultural difference in interpersonal communication might cause interactions between foreign teachers and Chinese people to remain at the level of superficial politeness without providing realistic emotional support. Very few foreign teachers could be accepted by Chinese colleagues or friends as “acquaintances” (Cao, 2017; Ma, 2007). In our study, this interpersonal style of communication even led to loneliness, feelings of poor ego strength, and lack of self-control among the participants. This finding implied the necessity to pay attention to the interpersonal isolation of foreign teachers because previous studies of liquid society have found that a long-term low sense of self-worth and an internal sense of loss of control may hinder an individual’s adult identity (Formica et al., 2017).

When coping with the challenges mentioned above, most foreign teachers adopted strategies such as trying to interact more with local people, seeking help from bilingual colleagues and students, maintaining connections with the culture of origin and with compatriots, and visiting a psychotherapist (e.g., P3, P4, P7, and P8). The common feature of these coping styles is the establishment of interpersonal connections providing mutual understanding and emotional and instrumental support. This finding suggests that supportive interpersonal relationships may play an important role in the process of cross-cultural adaptation for foreign teachers (Melby et al., 2008; Lo Coco et al., 2018). Other participants gathered information by carefully observing the surrounding environment to improve the efficiency of their cultural adaptation. This finding suggests that being sensitive to new information may be an effective strategy for foreign teachers’ adaptation to Chinese culture (Meng, 2017). Additionally, the common use of mobile apps by the participants suggested that apps might be effective tools to promote foreign teachers’ cultural adaptation.

In addition, some foreign teachers (e.g., P3 and P4) chose relatively fixed living habits, retaining some of their old living habits and maintaining regular connections with their culture of origin and compatriots, to reduce the stress of cross-cultural adaptation. The participants’ use of both this strategy and the functional interaction coping strategy suggests that relative stability and certain changes may be equally important for the cross-cultural adaptation of foreign teachers. In other words, attention should be paid to the connection with the culture of origin as well as to the acceptance of and participation in the current new culture (Melby et al., 2008; Meng, 2017). This opinion is in line with the “integration” model in Hui and Berry’s theory of cultural adaptation, which emphasizes the integration of local customs from the new culture and the motherland culture (Berry et al., 2002; Hui et al., 2015).

In contrast, some participants responded to challenges and emotions using insulation. Interestingly, we found that subjects with avoidant personality traits (e.g., P2 and P4) were more inclined to adopt this coping strategy. In addition, these foreign teachers seemed to be more vulnerable to negative emotional experiences such as embarrassment and anger. This finding suggests that foreign teachers’ personality characteristics and their automatic coping styles may both play important roles in their cultural adaptation (An and Chiang, 2015; Saffari et al., 2017). Nevertheless, how the interactions between those two variables influence the link between the challenges of culture adaptation and subjects’ outcomes, such as emotional status, still needs to be explored in future research. Moreover, according to feedback from the foreign teachers in our study, insulation allowed some participants to avoid the discomfort of excessive negative emotions. However, it seemed that too much suppression of emotional expression also limited their efficient adaptation to Chinese culture. This contradiction implies the value of exploring a more flexible and balanced usage of insulation as a strategy for managing negative emotions during cultural adaptation (An and Chiang, 2015). Hence, more research may be required to investigate how frequently foreign teachers should use insulation to protect themselves from excessive negative emotions while avoiding the occurrence of psychosomatic disturbance due to oversuppression of the expression of negative emotions (Liu et al., 2017a, b).

Differences in privacy and boundary concepts in interpersonal communication were also among the main challenges faced by foreign teachers. Our analysis suggested that sensitivity and the definition of privacy protection among foreign teachers and Chinese people differed, and this difference led to corresponding emotional reactions and conflicts. Aside from isolation, the participants in this research did not seem to have developed any other effective coping strategies. Interestingly, the excessive attention foreign teachers received from local Chinese residents seemed to make them uncomfortable, but also became a resource, as our analysis suggested, that their identities as foreigners caused teachers to receive special treatment on some occasions. However, follow-up research and exploration are needed to determine how to better use this “foreign” identity to promote foreign teachers’ adaptation to life in China.

With respect to conflicts related to teaching modes, the inducing factors may vary. On the one hand, conflict may arise due to Chinese teachers’ use of the one-way transmission method in education and the tradition of attaching importance to curriculum content and academic achievement. On the other hand, conflict may also occur due to the traditional Chinese Confucian cultural emphasis on seniority and the use of caution when speaking and acting in the presence of authority figures such as teachers (Melby et al., 2008; Cao, 2017). Interestingly, we found that some of the foreign teachers adjusted the interaction mode in their classes, the grade evaluation system, and the use of multimedia in teaching in line with the habits of Chinese students to resolve this conflict. These localized methods provide a reference for other foreign teachers who need to address similar problems in the future. Moreover, this finding also suggests that, for both foreign teachers and Chinese students, there are possibilities to integrate Western and Eastern teaching models, such as moving from traditional didactic teaching to more self-directed learning, to meet the requirements of a modern vocational education system (Ho and Tran, 2018).

When facing the above challenges, foreign teachers mainly felt emotions such as frustration, embarrassment, and anger, and these emotions were rarely expressed, understood, or processed. This result has rarely been reported in previous literature. Our analysis also demonstrated that these emotions were related not only to the challenge of adaptation itself but also to some of the foreign teachers’ negative orientations and self-deprecation. This suggests that it may be necessary to pay attention to and address the negative emotions that arise during foreign teachers’ adaptation processes from three angles: personal characteristics, practical challenges, and strategies for coping with stress.

Three other core themes emerged in our study: mixed negative emotions, supportive resources, and personal/family-of-origin traits. Our analysis indicated that these themes may have interacted with each other and with the first two themes during the subjects’ adaptation. The participants primarily mentioned diverse resources that facilitated their adaptation processes. However, we found that not all the participants adopted these resources. For example, although the results suggested that help from local Chinese people, smartphone apps, and convenient living facilities played important roles in foreign teachers’ adaptation, there were participants who were not used to using these facilities, especially those who had more personality traits associated with avoidance coping, low flexibility, and negative thinking (e.g., P2 and P6). Meanwhile, it seems that the teachers were more inclined to adopt dysfunctional coping strategies such as emotional insulation, self-sealing, and maintaining a fixed lifestyle. Thus, their ignorance of resources and adoption of avoidant coping may have made them more vulnerable to negative emotions (Liu et al., 2017a, b) and may have contributed to the creation of more challenges in their lives. This vicious interactional cycle among challenges, emotional response, coping, and personality traits might constitute the main mode of the maintenance of the subjects’ cultural adaptation difficulties. This is partially consistent with findings from previous research that maladaptive personality domains such as avoidant personalities and not-otherwise specified personality traits (low flexibility) were linked with diverse forms of emotional dysregulation (Cox et al., 2011; Dimaggio et al., 2012; Koenigsberg et al., 2019).

In contrast, for some other foreign teachers (e.g., P1, P3, P7, and P8), a more virtuous cycle appeared. These teachers were more inclined to exhibit positive thinking, to evaluate themselves as worthwhile, and to be more willing to interact with the people around them and adopt accessible resources. As demonstrated by the results, this functional interaction strategy not only improved foreign teachers’ cultural adaptation experience but also made their adaptation smoother. They were more inclined to regard difficulties as challenges and opportunities, and they experienced more positive emotions. This finding is partially consistent with the idea of cognitive behavioral therapy that the way individuals think about problems sometimes plays an important role in coping with problems (Ung et al., 2015; Jacobs et al., 2016; Wergeland et al., 2016). The finding also suggests that, when working with foreign teachers in psychotherapy, attention could be paid to exploring and changing the client’s perspective and coping models.

Additionally, regarding the resources supporting the subjects’ cultural adaptation, help from local agencies and local people in China as well as stable intimate relationships played an important role in foreign teachers’ adaptation, suggesting that interpersonal relationships provided ample emotional communication, mutual understanding, and instrumental support, which were factors that allowed foreign teachers to more smoothly complete their cross-cultural adaptation processes in China. This finding suggests that Chinese colleges and universities should consider establishing interpersonal connection programs or services that could provide more emotional and instrumental support for foreign teachers. Furthermore, similar to the views of Li (2012), this study found that foreign teachers’ cross-cultural life experiences and open attitudes toward multiculturalism could help them better adapt to Chinese culture and increase the flexibility of their coping strategies. Moreover, similar to previous studies, this study found that the participants who were enthusiastic about the Chinese culture had a more positive experience of adaptation in the country (Wang, 2010; Duan, 2012). Therefore, it may be possible to convey more positive information about China to foreign teachers through the media or other means before they arrive to create positive expectations about their lives in China and reduce their cross-cultural adaptation conflicts, homesickness, and culture shock (Duan, 2012; Li, 2012).

This study has several limitations. We did not distinguish between different periods of cross-cultural adaptation when analyzing the experience of foreign teachers. As Hottola (2004) mentioned, the process of cross-cultural adaptation occurs in different stages, during which visitors show various reactions. The experience shared by the participants in our study might be integrated with feelings from different adaptation periods. Thus, more research exploring foreign teachers’ experience during different stages of cultural adaptation is strongly suggested. Additionally, special attention was not paid to some factors that may affect foreign teachers’ adaptation, including attitudes, emotions, and perceptions regarding Chinese colleagues and government departments. These variables might also influence the process of cultural adaptation among foreign teachers (Cao, 2017). Hence, one possible suggestion for future research is to explore the ideas of foreign teachers’ families, friends, or Chinese colleagues. Finally, although some potential correlations among different themes and the influence of their interactions on foreign teachers’ adaptation experience emerged, due to the limited sample, the mechanism by which these factors interact with each other remains unclear. Future research with a larger sample to explore this topic is strongly suggested.

## Conclusion

Our analysis demonstrated that during the process of cross-cultural adaptation in China, the main challenges for foreign teachers were language barriers; differences in living habits; a lack of supportive relationships; and conflicts in perceptions related to teaching, privacy, and boundaries. When coping with those challenges and the corresponding negative emotions, useful strategies frequently adopted by the participants included seeking interpersonal interactions that provided active emotional and instrumental assistance, developing localized and integrated teaching models, maintaining connections with the culture of origin, and being sensitive to new information. Under circumstances with convenient living facilities and supportive interpersonal relationships, these methods promoted the cultural adaptation of foreign teachers who had cross-cultural life experiences, stable intimate relationships, higher psychological resilience, and positive attitudes. The themes that emerged in our study can help clinicians, university administrators, and other relevant parties develop more comprehensive, humanized, empathetic, and detailed therapy methods and support systems for foreign teachers who work in Chinese universities.

## Data Availability Statement

The datasets generated for this study are available on request to the corresponding author.

## Ethics Statement

The studies involving human participants were reviewed and approved by the Tongji University and Wenzhou-Kean University. The patients/participants provided their written informed consent to participate in this study.

## Author Contributions

XX and NW conducted interviews with the participants and transcribed the data. LL and SY developed the interview outline and took the main responsibility in drafting the manuscript. All authors contributed to the recruitment of patients and analyzed the data.

## Conflict of Interest

The authors declare that the research was conducted in the absence of any commercial or financial relationships that could be construed as a potential conflict of interest.
